# Effectiveness and safety of a new dental plaque removal device utilizing micro mist spray for removing oral biofilm in vitro

**DOI:** 10.1186/s12903-021-01647-4

**Published:** 2021-06-04

**Authors:** Hiroki Hihara, Ryo Tagaino, Jumpei Washio, Kittipong Laosuwan, Dimas Prasetianto Wicaksono, Kuniyuki Izumita, Rie Koide, Nobuhiro Takahashi, Keiichi Sasaki

**Affiliations:** 1 Division of Advanced Prosthetic Dentistry, Tohoku University Graduate School of Dentistry, 4-1 Seiryo-machi, Aoba-ku, Sendai, Miyagi 980-8575 Japan; 2 Division of Oral Ecology and Biochemistry, Tohoku University Graduate School of Dentistry, Sendai, Miyagi Japan; 3 Department of Oral Biology and Oral Diagnostic Sciences, Faculty of Dentistry, Chiang Mai University, T. Suthep, A. Muang, Chiang Mai, 50200 Thailand; 4 Faculty of Dental Medicine, Department of Pediatric Dentistry, Universitas Airlangga, St. Mayjen Prof. Dr. Moestopo No. 47, Surabaya, 60132 Indonesia

**Keywords:** Oral care, Oral biofilm, Micro scale mist, Oral mucosa, Translational research

## Abstract

**Background:**

Removal of oral biofilm from the oral mucosa is essential for preventing risk of respiratory and gastrointestinal infection in elderly people. Currently, no device is available which can remove oral biofilm from oral mucosa effectively and safely. Therefore, the effectiveness and safety of the Micro Scale Mist UNIT (MSM-UNIT), a newly developed dental plaque removal device utilizing high speed sprays of fine water droplets, were evaluated for biofilm removal, including the rate and surface roughness for simulated tooth surface and mucous membrane.

**Methods:**

Simulated tooth and oral mucosa coated with an artificial biofilm of *Streptococcus mutans* were used for evaluation of effectiveness, with uncoated substrates as the controls. The MSM-UNIT and a conventional air ablation device were operated under recommended instructions. The effectiveness was evaluated from the rate of removal of the biofilm, and the safety was evaluated from the damage observed by scanning electron microscope and surface roughness.

**Results:**

The biofilm removal rate of the MSM-UNIT was significantly higher than that of AIRFLOW. Little damage was observed in the area treated by the MSM-UNIT. The surface roughness of the MSM-UNIT treated area on simulated tooth surface and oral mucosa showed no significant difference to the control area. In contrast, cracks and powder were observed in the area treated by AIRFLOW. In particular, the surface roughness of the AIRFLOW treated area for Toughsilon was significantly larger than that of the control.

**Conclusions:**

The MSM-UNIT could be used safely and effectively for removing biofilm not only on simulated tooth surfaces but also simulated mucous membrane. The MSM-UNIT has no harmful effect on teeth or oral mucosa, and may be used for comprehensive oral care for patients during nursing care and the perioperative period.

## Background

Oral biofilm such as dental plaque is a mass of bacteria and their polysaccharide metabolites firmly attached to the tooth surfaces, tongue, and cheek mucosa. Oral biofilm is the cause of dental caries and periodontal disease [1] and also a cause of aspiration pneumonia, endocarditis, and fever [1, 2]. Removal of oral biofilm helps to prevent respiratory infections and gastrointestinal infections in elderly people requiring long-term nursing care [3–5], as well as reduce the infection risk of hospitalized patients [6], shorten the period until discharge, and improve the quality of life [7]. However, current cleaning methods based on toothbrushes and auxiliary cleaning tools such as water jets [8] requires training and skill of the patients and caregivers. In addition, biofilm forms on the oral mucosa [9]. In particular, oral membranous substances were frequently observed in bedridden elderly persons without oral intake and with nursing care, being composed of inflammatory cells and bacteria [10–12]. It can be considered that these substances are types of biofilm and difficult to remove.

While air flow devices, which deliver powders of hard material and water through air flow, have been developed to treat gingivitis and periodontitis [13–19], they are only approved for cleaning dental biofilm on tooth surfaces and around periodontal tissue. No approval has been obtained for cleaning oral biofilms on the entire oral mucosa of patients. Cleaning of adhered oral biofilm on the oral mucosa is associated with risk of damaging the mucosa and aspiration, and thus taxing to both physical and mental stress of caregivers.

**Fig. 1:**
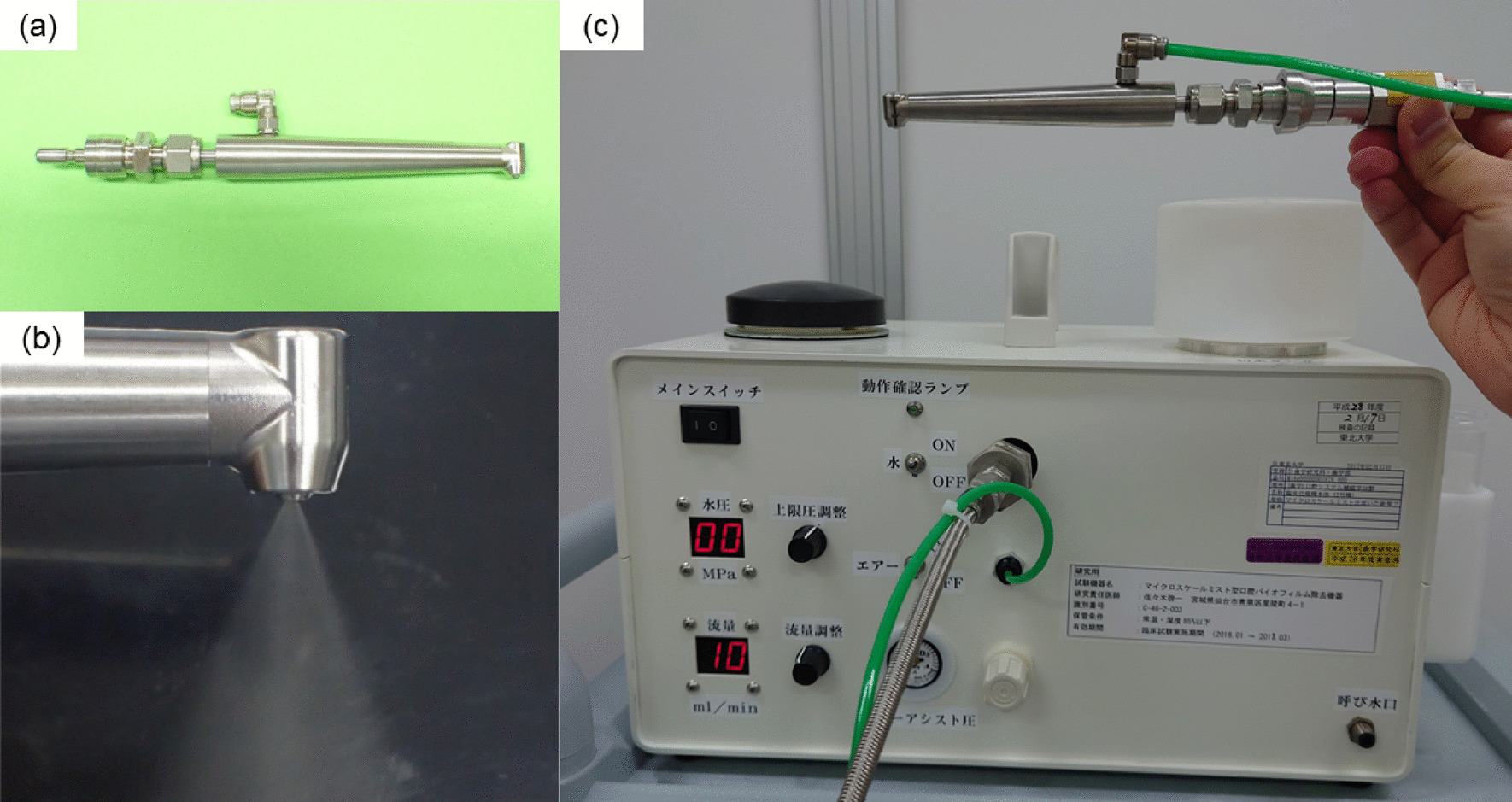
Photographs of our device named “Micro Scale Mist UNIT” (MSM-UNIT) showing the handpiece (**a**), spraying with the handpiece (**b**), and the main body of the MSM-UNIT (**c**)

Recently we developed a technique for the simple and safe removal of oral biofilm based on fine water droplets sprayed at high speed (few m/sec) onto the tooth surface and oral mucosa [20]. Water droplets of average diameter 40 μm were produced at the outlet of the handpiece from water pumped at high pressure (2 MPa) and blasted through the nozzle with the aid of air pressure (9 MPa). The greatest advantage of this technology is the removal of biofilm at low power to avoid mucosal pain and injury, as water droplets with micro diameter (about 40 μm) have extremely low mass despite their high kinetic energy. Our device operates on markedly different principles to the high-pressure water flow of a conventional water jet, and so has no harmful effect to either the teeth or the oral mucosa. We have named our device the “Micro Scale Mist UNIT” (MSM-UNIT) (Fig. 1). Fluid science study has revealed that the dominant mechanism of artificial plaque removal is most likely the kinetic impact of fine droplets displacing the biofilm more so than shear stress breaking the coating [20]. The aim of our study was to obtain clinical approval of MSM-UNIT for use as a medical device. The first step to obtaining approval is the evaluation of the effectiveness and safety of the device for not only teeth but also oral mucosa in vitro, which were the main focuses for designing this device.

Therefore, this study evaluated the effectiveness and safety of the MSM-UNIT device in vitro, using an artificial biofilm model on a simulated tooth surface and oral mucosa.

## Methods

### Fabrication of artificial tooth and mucosa model

Slide glass (Aslab Super White Glass Micriscope Slides, ASONE, Osaka, Japan) was used as the simulated tooth surface model. Toughsilon (TSG-E10, TANAC Co., Ltd., Gifu, Japan) and Sofreliner (Medium Soft, Tokuyama Dental Corp., Tokyo, Japan) bonded onto plastic slide glass (Plastic slide glass, KENIS LIMITED, Osaka, Japan) were used as the simulated oral mucosa model.

Toughsilon was chosen for modeling relatively soft regions, such as buccal and the soft plate, and Sofreliner for relatively hard regions, such as the back of the tongue and palate. The elastic moduli measured by the softness sensor SOFTGRAM (SHINKO DENSHI CO., LTD, Tokyo, Japan) were 1.4 MPa for Sofreliner and 0.4 MPa Toughsilon gel, respectively. The elastic moduli of the mucous membrane reported in the literature is 2.42 ± 0.84 MPa on average for hard regions and 0.98 ± 0.49 MPa for soft regions [21]. Therefore, the chosen materials had similar elastic moduli and could be obtained easily, aiding the reproducibility of the experiment.

Slide glass was sterilized in an autoclave. Toughsilon gel and Sofreliner were disinfected by immersion in 76.7–81.4 volume% ethanol (FUJIFILM Wako pure chemical corporation, Osaka, Japan) for a few minutes. These samples were used as the base for the biofilm formation.

Since the biofilm in the oral cavity adheres to the tooth and mucosal surfaces via the pellicle formed of saliva, the simulated tooth and oral mucosa surfaces were also coated with saliva in this study. Saliva for coating was collected from three healthy volunteers. This sampling method was approved by the ethical committee of the Tohoku University Graduate School of Dentistry (protocol number: 2018-3-017) in accordance with the Declaration of Helsinki. Written informed consent was obtained from all the healthy volunteers. The saliva was centrifuged at 10,000 rpm for 7 min and the supernatants were filtrated with a 0.22 μm sterile filter (Millex-GV Syringe Filter Unit/0.22 μm/gamma sterilized, Merck KGaA, Darmstadt, Germany). Both simulated tooth and oral mucosa surface models were coated with this filtered saliva at 4˚C overnight, and then washed twice softly with sterilized physiological saline to remove the saliva.

Artificial biofilm was formed with *Streptococcus mutans* NCTC 10449 (SM), a representative dental caries-related bacteria known to produce insoluble glucan from sucrose and adhere easily and strongly to surfaces. SM was maintained on blood agar plate (Nippon BD, Tokyo, Japan). For culturing SM and forming biofilm, we modified the general culture medium for SM [22, 23] to one containing 1.7 % tryptone, 0.3 % yeast extract, 0.5 % NaCl, 50 mM potassium phosphate buffer (pH 7.0) and 2 % sucrose (tryptone-yeast extract-sucrose [TYS]).

The simulated tooth surface and oral mucosa models were immersed into a sterilized glass container filled with TYS. Biofilm formation was started by adding 1 % of pre-cultured SM, and incubated aerobically at 37 ˚C for 7days. TYS in the glass container was replace with a new one every 24 h. After confirming that biofilm had formed on the surfaces, these simulated models were separated from TYS and gently washed with sterilized physiological saline.

An identical mucosa model without biofilm was prepared for the safety evaluation. Safety for the simulated tooth surface (slide glass) is well known, and thus not evaluated in this study.

### Devices for biofilm removal

The MSM-UNIT and AIRFLOW® Prophylaxis Master (EMS Electro Medical Systems SA, Nyon, Switzerland) device were used for evaluating effectiveness and safety. AIRFLOW was used as a device for comparison, since it has acquired clinical approval for use around the periodontal tissue with erythritol powder. Water jet devices for the oral region do not have clinical approval as medical devices in Japan, where their main use is as auxiliary cleaning devices. Therefore, AIRFLOW was deemed more suitable for the comparison device. We attempted to remove the artificial biofilm on the simulated tooth and oral mucosa surfaces using each device under the following conditions.

### Application of MSM-UNIT

According to the recommended usage of MSM-UNIT [20], water mist was continuously sprayed vertically onto the surface of the samples at the same position for 10 s with a water flow rate of 10 ml/min, air assist pressure of 0.2 MPa, and distance from the surface of 10 mm. The affected area had a diameter of about 5 cm.

### Application of AIRFLOW® Prophylaxis Master

Erythritol powder (AIR-FLOW® Powder Plus; EMS Electro Medical Systems SA) suitable for the supragingival and subgingival area was sprayed continuously onto the samples at the same position for 10 s at a water flow rate of 10 ml/min, air pressure of 3 MPa, and distance from the surface of 5 mm at an angle of 30° to 50°, in accordance with recommended usage. The affected area had a diameter of about 5 cm.

### Evaluation methods

#### Effectiveness

Standardized photographs were taken before and after treatment by microscope camera (U06; Kenis Co., Ltd., Taiwan) in a 2.5 × 3.5 cm region centered on the affected area. The imaging area was binarized by setting luminosity from 69 to 255 using image analysis software (DIPP-Image; DITECT Co., Ltd., Tokyo, Japan) to evaluate the effectiveness of biofilm removal. Biofilm adhesion area was calculated and the biofilm removal rates (%) determined through the following formula: (1 – biofilm adhesion area after spraying/ biofilm adhering area before spraying) × 100. The biofilm removal rate was compared between samples, using t-test for statistical comparisons. Differences were considered significant at *p* < 0.05.

#### Safety

Damage to the samples without biofilm were evaluated by scanning electron microscope (SEM) (SU500; Hitachi High-Tech Corp., Tokyo, Japan). Surface roughness (Ra) was calculated using the SEM analysis software (OIM EBSD System; AMETEK, Inc., Kent, OH, USA). Ra was measured at three locations for each sample. Ra of the non-affected area (control), MSM-UNIT affected area, and AIRFLOW affected area were compared. Dunnett’s test was used for statistical comparisons. Differences were considered significant at *p* < 0.05.

## Results

### Effectiveness

**Fig. 2:**
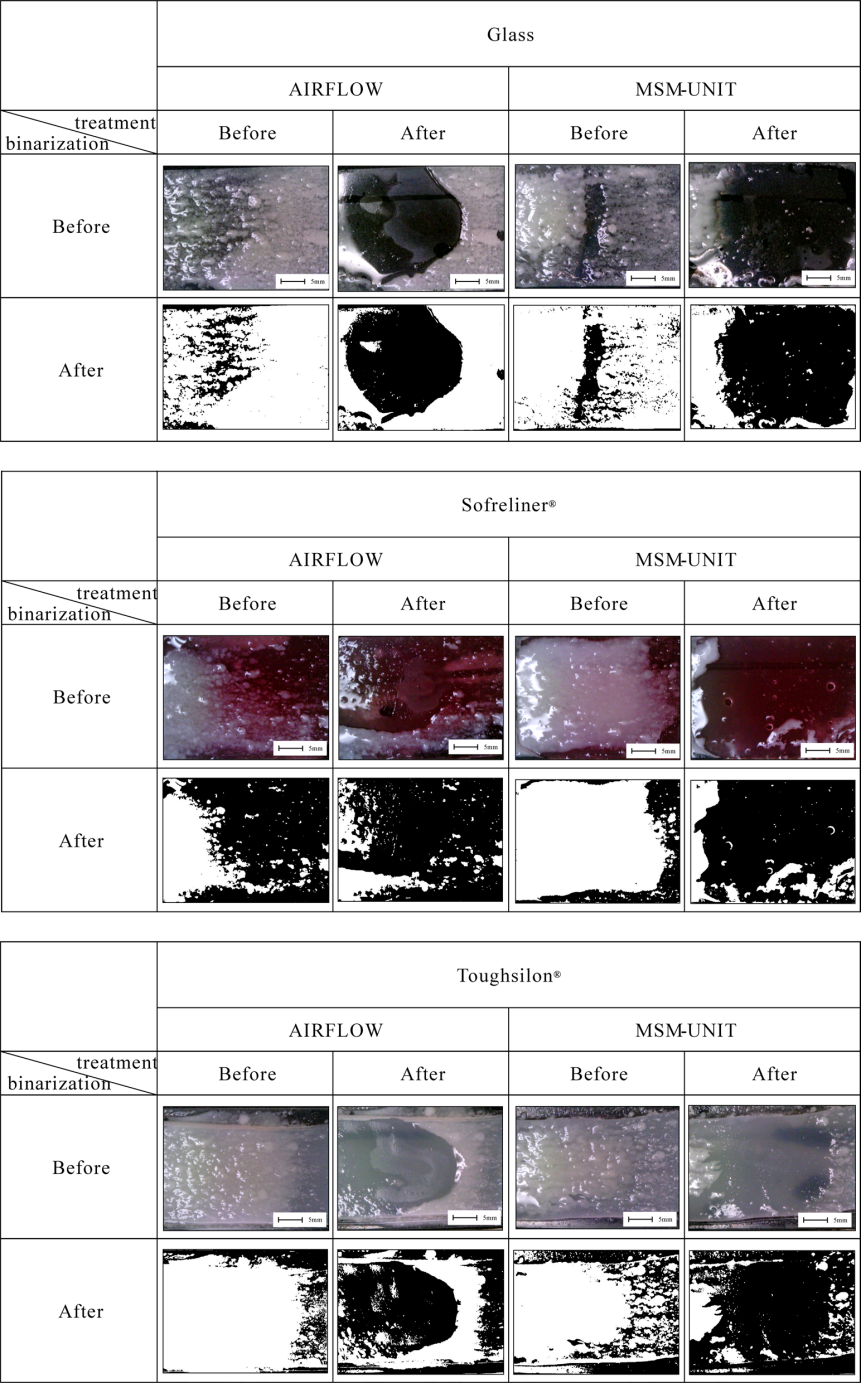
Simulated tooth and mucosa model with biofilm before and after spraying by AIRFLOW and MSM-UNIT. Photographs before and after the binarization are shown

Effectiveness evaluations are shown in Fig. 2. The removal rate of biofilm (mean ± SD %) by the MSM-UNIT was 60.0 ± 12.8 % on slide glass, 68.3 ± 6.6 % on Sofreliner, and 71.2 ± 3.4 % on Toughsilon. The removal rate of biofilm by AIRFLOW was 32.5 ± 9.9 % on slide glass, 51.8 ± 8.5 % on Sofreliner, and 48.5 ± 8.3 % on Toughsilon. The removal rates on slide glass and Toughsilon by the MSM-UNIT were higher than that by AIRFLOW (*p* < 0.05). Removal rates of biofilm on Sofreliner showed no significant difference between the two devices.

### Safety

**Fig. 3:**
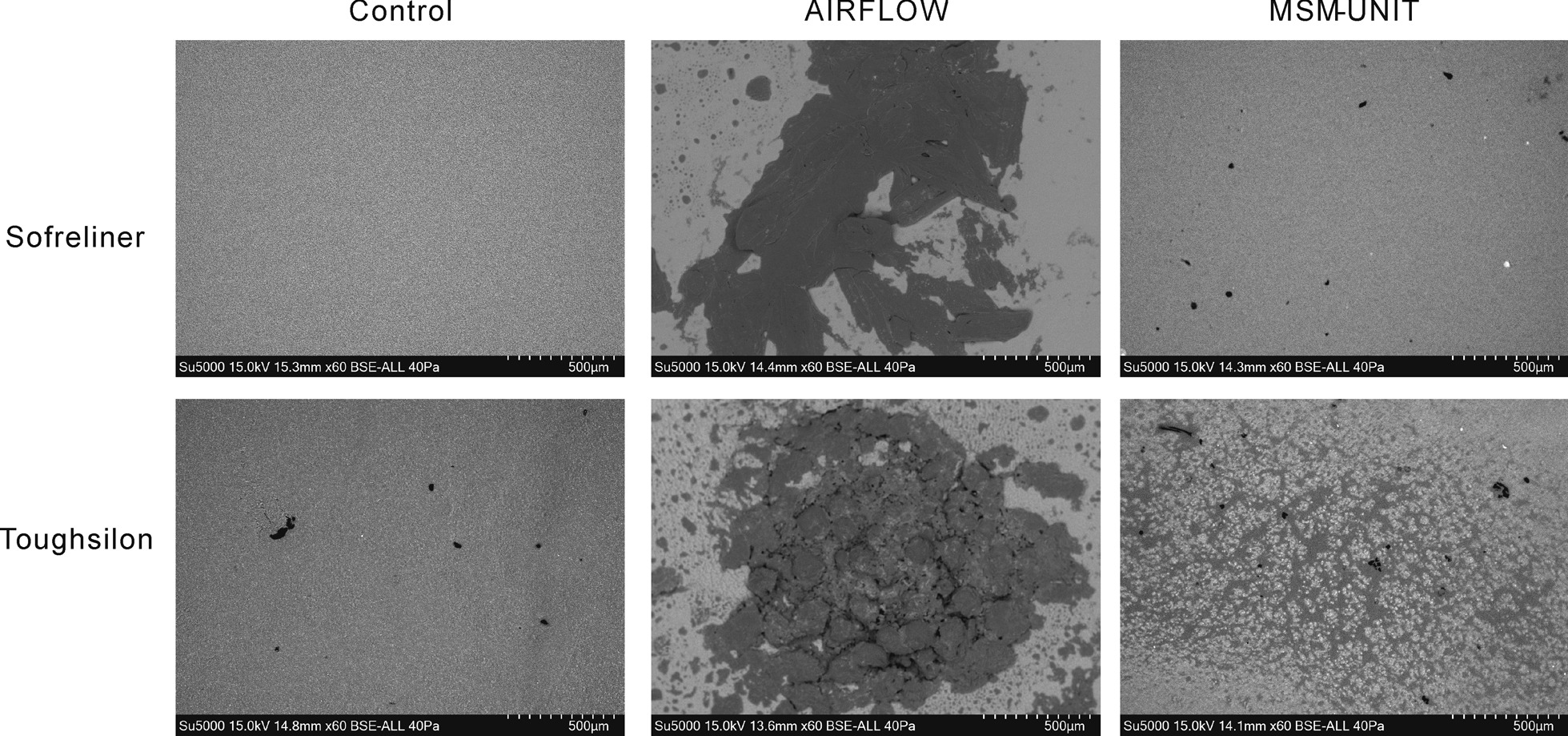
SEM images of Sofreliner and Toughsilon simulated oral mucosa, control and after 10 s treatment by AIRFLOW and MSM-UNIT

SEM images are shown in Fig. 3. Simulated oral mucosa of Sofreliner and Toughsilon treated using AIRFLOW showed rough surfaces with large damaged spots, whereas those using MSM-UNIT had relatively smooth surfaces only with small damaged spots. The Ra of the control area was 58.7 ± 0.7 μm on Sofreliner and 65.5 ± 0.9 μm on Toughsilon. The Ra of the MSM-UNIT treated area was 57.0 ± 8.1 μm on Sofreliner and 66.2 ± 8.1 μm on Toughsilon (Table 1). The Ra of the AIRFLOW treated area was 66.2 ± 7.3 μm on Sofreliner and 90.2 ± 23.5 μm on Toughsilon. The Ra of the AIRFLOW treated area on Toughsilon was significantly larger than that of the control. In contrast, Ra of the control showed no significant difference to the MSM-UNIT or AIRFLOW treated areas on Sofreliner.

**Table 1:** Biofilm removal rate and surface roughness using AIRFLOW and MSM-UNIT

Device	N	Biofilm removal rate (%)			Surface roughness (µm)	
		Glass	Sofreliner	Toughsilon	Sofreliner	Toughsilon
AIRFLOW	3	32.5 ± 9.9	51.8 ± 8.5	48.5 ± 8.3	66.2 ± 7.3	90.2 ± 23.5*
MSM-UNIT	3	60.0 ± 12.8*	68.3 ± 6.6	71.2 ± 3.4*	57.0 ± 8.1	66.2 ± 8.1

Values are mean ± SD. The biofilm removal rate of the MSM-UNIT was significantly different to that of AIRFLOW (*p < 0.05). The surface roughness of AIRFLOW for Toughsilon was significantly larger than that of the MSM-UNIT (*p < 0.05). The surface roughness of the AIRFLOW treated area for Toughsilon was significantly larger than that of the control

## Discussion

This study indicates that the MSM-UNIT was effective for the removal of biofilm on the simulated tooth surface and oral mucosa. The high removal rate achieved by the MSM-UNIT is partly explained by the spray direction of water droplets onto the substrate and the behavior of water after the collision with the substrate. Since the MSM-UNIT sprayed relatively perpendicular to the substrate, the water droplets colliding with the substrate spread circumferentially and pushed the biofilm aside in various directions. In contrast, the AIRFLOW powder was sprayed at an angle of nearly 45°, and pushed the biofilm aside in only one direction.

Biofilm removal is achieved by high flow shear stress levels that exceed the adhesion strength of the biofilm [24]. Previous studies showed that 5–12 Pa of shear stress is required for removal of non-dental biofilms [24, 25]. The thick biofilm formed by *S. mutans* can be removed by relatively a low shear stress of about 2 Pa [26]. The shear stress generated by the MSM-UNIT was estimated at 10.5 kPa, which is adequate to remove dental biofilm. Additionally, the main mechanism of biofilm removal for the MSM-UNIT was the impact of droplets displacing the plaque, the pressure of which was estimated at 2 × 10^8^ Pa [20], greatly exceeding the required shear stress. Therefore, the MSM-UNIT has sufficient capacity to remove biofilm.

The AIRFLOW device caused cracks and powder deposits on the simulated oral mucosa as shown in Fig. 3. The Ra of the AIRFLOW treated area for Toughsilon was significantly larger than that of the control, while that for Sofreliner did not significantly differ. Since Toughsilon is softer than Sofreliner, the surface of Toughsilon was damaged by AIRFLOW treatment, resulting in an increase in Ra. On the other hand, the MSM-UNIT caused only few cracks on the simulated oral mucosa, and no significant differences in the Ra of the MSM-UNIT treated area for Sofreliner and Toughsilon were found compared to the controls. Therefore, the MSM-UNIT was demonstrated to be safer for use on oral mucosa than air abrasion devices such as AIRFLOW.

Effective oral care reduces the incidence of aspiration pneumonia in nursing homes and hospitals [3–6]. Candida-associated denture stomatitis is a frequent oral infection that affects up to 60 % of denture wearers and causes inflammation of the palatal tissues [27]. Use of a sponge brush for oral biofilm removal on oral mucosa has been shown to reduce *Candida albicans* [28]. The number of bacteria on the oral mucosa is reduced by chlorhexidine gargle [29]. Additionally, bacteria in the pharynx are reduced by wiping the oral mucosa with a sponge brush soaked in chlorhexidine [30]. Therefore, removal of biofilms on the oral mucosa is definitely important for patients in nursing homes and hospitals. However, using a sponge brush efficiently requires proper technique, and chlorhexidine has adverse effects such as allergies, soreness, irritation, mild desquamation, and mucosal ulceration/erosions [31]. On the other hand, for the MSM-UNIT carries almost no risk of allergies since only water is used, leaving only aspiration as a remaining concern. Compared to the water flow rate of a turbine of 60–70 ml/min, the rate of the MSM-UNIT is much lower (10 ml/min), and so can be used safely with a usual suction device.

New methods for removing oral biofilm using plasma jet [32], ultrasonic activated water [33, 34], and water jet [8] have been shown to be effective. However, application of these devices is limited only to teeth, and the effect on the oral mucosa is unknown. The present study thus adopted air-polishing devices for comparison, which use glycine powder to remove biofilm in the gingival area [35]. Patients report high satisfaction from these devices [36]. Correct use does not cause harmful effects to the oral mucosa [37]. However, the simulated mucosa material employed in this study, which was softer than the oral mucosa, suffered slight damage using Erythritol powder. Additionally, much powder is consumed by these devices and extraoral suction is required. On the other hand, the MSM-UNIT did not affect the simulated mucosa, and so is a simpler and safer method for the removal of oral biofilm. The MSM-UNIT can be used for elderly people and patients in nursing homes and hospitals, who are expected to increase worldwide in the future, and also enables comprehensive plaque removal from the oral mucosa. However, in this study, a simulated mucosa with similar hardness to the oral mucosa was selected, and its water retention rate differed to that of the actual oral mucosa. In addition, the artificial biofilm on oral mucosa was slightly different to actual oral biofilm, so more detailed evaluation is needed. Therefore, as a next step clinical study will confirm the safety and effectiveness of the MSM-UNIT.

## Conclusions

The MSM-UNIT was able to remove artificial biofilm on both simulated tooth surface and simulated oral mucosa, with higher efficiency and safety than an AIRFLOW device. Further human studies including clinical trials are needed to assess the effectiveness and safety of the MSM-UNIT for use in patients during nursing care and the perioperative period.

## Data Availability

The datasets used and/or analyzed during the current study are available from the corresponding author on reasonable request.

## Abbreviations

MSM-UNIT: Micro Scale Mist UNIT
Ra: Arithmetic average roughness
SEM: Scanning electron microscope
TYS: Tryptone-yeast extract-sucrose
